# Physical, morphological, and wound healing properties of a polyurethane foam-film dressing

**DOI:** 10.1186/s40824-016-0063-5

**Published:** 2016-06-04

**Authors:** Seung Moon Lee, Il Kyu Park, Yong Soo Kim, Hyun Jung Kim, Hanlim Moon, Stefan Mueller, Young-IL Jeong

**Affiliations:** Genewel Co. Ltd., Gyeonggi-do, Korea; Mundipharma Pte. Ltd., Singapore, Singapore; Biomedical Research Institute, Pusan National University Hospital, 179 Gudeok-ro, Seo-gu, Busan, 602-739 Republic of Korea

**Keywords:** Wound healing, Porosity, Foam dressing, Absorption/retention capacity, Absorption pattern, Moisture-vapor transmission rate

## Abstract

**Background:**

We investigated the physicochemical properties of Medifoam® N and its wound healing performance compared to other commercially available polyurethane (PU) foam dressing in vitro and in vivo to gain insight in their clinical performance.

**Methods:**

Wound contact layer and cross-section of eleven polyurethane foam dressings were assessed with field-emission scanning electron microscope. Thickness, density, tensile strength, elongation, moisture-vapor transmission rate (MVTR), retention and absorptivity were measured to compare physical properties. Phosphate-buffered saline (PBS) solution absorption patterns were compared. An animal model for wound-healing was applied to validate in vitro findings.

**Results:**

Among eleven tested foam dressings, Medifoam® N has the smallest pore and cell sizes with excellent uniformity, i.e. it has 25 ~ 75 μm on the wound contact layer and 100 ~ 350 μm in the cross-section while other dressings have a larger pose size with larger variability. Compared to other PU foams, Medifoam® N also has moderate thickness, density, tensile strength, elongation and MVTR. Furthermore, it has excellent fluid absorption and retention capacity. These intrinsic properties of Medifoam® N contributed to improve fluid absorption patterns, i.e. other dressing material flawed out PBS solution on the dressings while Medifoam® N retained all the tested solutions. In animal wound-healing study, Medifoam® N treated animals showed excellent angiogenesis and collagen deposition even though epithelial recovery rate was not significantly different to other dressings.

**Conclusions:**

Medifoam® N has optimized physical properties and thus improved fluid absorption/retention capacity. Compared to other dressings, Medifoam® N showed excellent fluid absorption patterns and these characteristics contributed to improved wound healing and excellent angiogenic potential. We found that Medifoam® N showed the best results among the employed dressing samples.

**Electronic supplementary material:**

The online version of this article (doi:10.1186/s40824-016-0063-5) contains supplementary material, which is available to authorized users.

## Background

Key objectives in wound healing are the reduction of infection and pain as well as the promotion of tissue repair [1–3]. Various kinds of materials have been used to this purpose. In ancient times, natural materials including honey pastes, plant or animal materials, and cloth have been used for wound healing purposes [1, 2, 4]. In the last several decades, novel materials using synthetic biocompatible polymers and natural polymers have been developed to improve the wound healing performance [4–8]. Natural polymers such as chitin and chitosan have been extensively investigated since they have adhesive properties, antifungal/antibacterial characters, and oxygen permeability [6]. Synthetic polymers were also investigated for wound healing purposes [4, 6, 7]. For example, polypeptide-poly(ethylene glycol) block copolymer was reported to have suitable properties for wound healing with suppression of bacterial proliferation, granulous tissue formation and wound contraction [7].

**Scheme 1:**
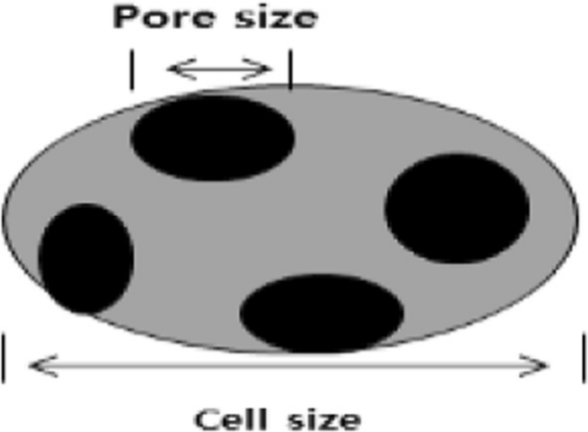
Pore size measurement from FE-SEM observationScheme: Scheme “1” was received; however, no citation was provided in the manuscript. Please provide the location of where to insert the citation in the main body of the text. Otherwise, kindly advise us on how to proceed. Please note that schemes should be cited in ascending numerical order in the main body of the text.Scheme 1 should be moved to Experimental section (next to morphology observation).

In other words, physical properties of wound healing materials play a key role in creating an environment conductive to wound healing [4]. Junker et al., reported the importance of the microenvironment to wound healing [9]. They emphasized that a wet, incubator-like microenvironment is important to provide the fastest healing with fewest aberrations and least scar formation. Bilayered polyurethane wound dressing composed of microporous top layer and highly porous sponge-like sublayer are known to provide efficacy in the prevention of dehydration, bacterial penetration, and bullae formation [8]. Furthermore, Doillon reported the importance of porous structure on the wound tissue infiltration in vivo as well as cell growth in vitro [10]. The goal in selecting a suitable dressing is to create an optimal environment that best facilitates healing by providing protection from contamination and infection, enhancing the activity of enzymatic and cellular systems to promote re-epithelialization, and controlling the biomechanics of the wound area to provide mechanical stability [3, 11–13]. The use of inappropriate wound dressings can contribute to the development of infection or the formation of excessive scar tissue, which significantly undermines a proper healing process [3, 13]. Furthermore, exudate containment is regarded to an importance factor in the quality-of-life problems since excessive exudate leads to malodor and leakage resulting in loss of sleep, depression, and social isolation in affected patients [14].

In this study, we performed a wide range of laboratory tests using various commercially available dressings to evaluate their physical and morphological characteristics with respect to potential wound healing properties. This physicochemical testing may provide insight into the performance of medical devices, which essentially perform on physical, not pharmacological, metabolic or immunological modes of action. The relationship between physicochemical properties and biological/pathological performance of dressings was of particular interest. Additionally, an animal wound healing model was used to evaluate actual wound healing comparing traditional gauze dressing and various polyurethane foam dressings.

## Methods

### Materials

Medifoam® N was obtained from Genewel Co. Korea. Dressing A, B, A1, L, P, S, C, F, T, M, P1 were purchased from Allevyn (Smith & Nephew Co.), Biatain (Coloplast Co.), Askina (Braun Co.), Lyofoam Extra (ConvaTec Co.), Permafoam (Paul Hartmann Co.), Suprasorb (Lohmann & Rauscher Co. Ltd.), Cellosorb Adhesive (Urgo Medical Co.), Foam-S. (3 M Co.), Tegaderm (3 M Co.), Mepilex (Mölnlycke Health Care Co.), and Polymem (Ferris Manufacturing Co.), respectively. PBS (pH 7.4, 0.01 M) with pigment (SCU656, 0.1 %, DaeBo Co. ltd, Gyeonggi-do, Korea) was purchased from Gibco (NY, USA). All organic solvents were used as extra pure grade without further purification (Scheme 1).

### Morphology observation

Morphology of each dressing was assessed with field-emission scanning electron microscope (FE-SEM, S-4800, Hitachi, Tokyo, Japan). For FE-SEM testing, each dressing was cut into uniform size and then coated by platinum coater. Observation was performed at 25 kV. In the morphology analysis, the pore size and pore size uniformity of each dressing were measured and compared.

### Thickness and density measurements

Dressing thickness was measured with MDH micrometer high accuracy sub-micron digimatic micrometer (CD-15CPX, Mitutoyo Co. Ltd., Kawasaki, Japan). Thickness was measured at least 10 times with different samples and then expressed as average ± standard deviation.

For density measurement, width and length of each dressing was measured and then density was calculated as follows: Density(g/cm^3^) = weight/width × length × thickness. Density of each dressing were measured at least 10 times with different samples and then expressed as average ± standard deviation.

### Physical properties of dressings

All physical properties of dressings were estimated performed according to the methods described in American Society for Testing and Material (ASTM) or European Norm (EN) [15–17].

#### Moisture vapor transmission rate (MVTR)

The method for evaluation of MVTR was used the reported method by Khan et al., [5] with slight modification. Aluminum cup (diameter: 62 mm, absorptive area: 28 cm^2^) were pretreated in a dry oven (100 °C) 2 h prior to testing. Then, 20 g CaCl_2_ was put into this aluminum cup and then each dressing tied onto the top of the aluminum cup. 10 glass bottles were used for each dressing. 2 h prior to test, paraffin was pretreated in a dry oven (100 °C). In the aluminum cup, the specimen was set to face CaCl_2_ and the dead center of the cup to create a concentric ring. Melted paraffin was poured along the edge of the absorptive cup (beaker) to seal the edges. The paraffin debris around the joint cup was removed and then initial weight of the joint cup was measured (W_0_). The joint cup put into the thermo-hygrostat (37 °C, 75 % humidity) for 24 h, after which the weight of the joint cup was measured (W_24_). Then, the MVTR of the samples were calculated as follows:

$$ \mathrm{Moisture}\ \mathrm{vapor}\ \mathrm{transmission}\ \mathrm{rate}\ \left(\mathrm{g}/{\mathrm{m}}^2/\mathrm{hrs}\right) = \left[\left({\mathrm{W}}_{24}\hbox{-} {\mathrm{W}}_0\right)/0.0028*\mathrm{B}\right]*24\ \mathrm{h} $$

W_0_: initial weight of the sample before put into the thermo-hygrostat

W_24_: weight of the sample in thermo-hygrostat for 24 h

0.0028: unit conversion of absorptive area (cm^2^ → m^2^)

B: real time

#### Absorption rate and moisture retention capacity

Absorption rate was measured as follows: All dressings were cut into (5 × 5) cm and weighed (W1). Excess amount of deionized water (40 times higher than dressings, 37 ± 1 °C) was added 500 ml beaker. Temperature was controlled with pyrostat (OF-21E, Jeio Tech, Daejeon, Korea) at 37 °C for 30 min. After that, dressings were suspended and then weighed for 30 s (W2). The absorption rate was calculated as follows: [Absorption (g/cm^2^) = (W2-W1)g/Initial area of dressing (cm^2^).

Retention capacity was measured as follows: Initial weight (A) and thickness (B) of dressings were measured to similar with absorption study. All dressings were pressed with standard weight (111209, Jongro industrial Co., Ltd., Seoul, Korea) by 5 kg-weigh, for 20s and then calculated the retention weight (D) as follows: Retention capacity (g/cm^2^) = D – A/Initial area of dressing (cm^2^).

#### Tensile strength

To measure tensile strength, dressings were carefully cut into the form of Dog-bone (6 mm × 105 mm) using a wood-molded blade. The surface was kept unscathed when cutting. Tensile strength was measured with a material test machine (3343Q9831, Instron Co., MA, USA). Dressings were installed into the equipment symmetrically across the cross-section of the grip of the instrument and then dressings pulled out at a speed of 300 mm/min to measure the tensile strength at snap. Tensile strength was calculated as follows: [Tensile Strength (kgf/cm^2^) = MAX Failure Strength (kgf)/Cross-section of Specimen (mm^2^)]. Elongation was calculated as following equation: Elongation(%) = (D2-D1)/D1 × 100. D1: Initial Inter-clip Distance. D2: Inter-clip Distance at Snap.

### In vivo testing of wound healing

The wound healing abilities of dressings were studied by an animal wound model using Sprague Dawley (SD) rat (200 ~ 220 g, 8 week). All rats were freely fed water and food during experiment and 6 rats were used for each group. The backs of rats were shaved and then circular wounds with 25 mm diameter induced using a blade. Wounds were treated by foam dressings and then covered with gauze to avoid contamination, and secured with a film dressing (polyurethane film, Opsite, Smith & Nephew, UK). Dressings were bandaged to prevent nibbling by rats and replaced on 2 to 3-day intervals. General symptomatic properties, mortality rate, body weight, eating and drinking habits were comprehensively observed on a daily basis. Epithelialization of wounds on the backs of rats was assessed at days 3, 7, 10 and 14 by analysis of photographs. The sizes and epidermal recovery rate of wounds were measured based on observed photographs. At day 7, Tissues were collected for evaluation of impaired skin tissues and adapted with immunohistochemistry. All animal study was carried out according to the guidelines of committee of Genewel Institutional Animal Care and Use Committee (IACUC) (Ref. No: GAP-AVAL-14021).

Angiogenesis during wound closure was evaluated as following: At day 14, tissues were collected and dyed (hematoxylin and eosin (H&E) stain kit, American MasterTech, CA, USA) to assess angiogenesis in the newly forming skin tissue. Collagen deposition over wound care was assessed as the level of inner skin deposition and thickness were evaluated in assessment of full recovery of the impaired skin tissue.

## Results

### Analysis of morphological and physical properties

**Fig. 1:**
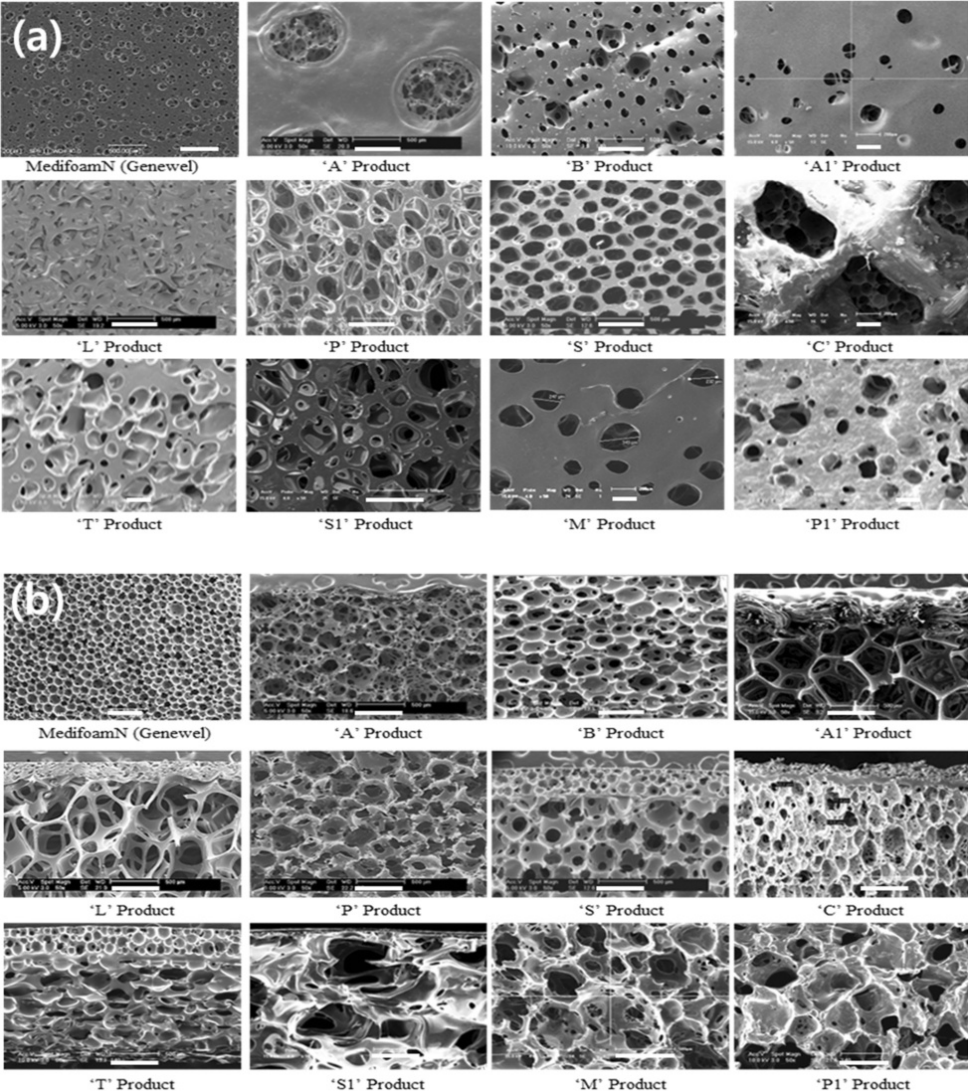
Morphological observation of surface (wound contact layer (**a**) and cross section (**b**) of dressings by FE-SEM

To observe morphological differences, surface (wound contact layer) and cross-section of dressings were observed with FE-SEM as shown in Fig. 1. As shown in Fig. 1a, Medifoam® N has relatively uniform and smaller pore size compared to other dressings, i.e. surface pore size of Medifoam® N was about 25 ~ 75 μm while pore size of other dressings were ranged from 32 to 1000 μm with lack of uniformity. Furthermore, cross-section of each dressing was also observed as shown in Fig. 1b. Except Medifoam® N, all dressings have non-homogeneous pore sizes and morphologies and pore sizes ranged from 169 μm to 1000 μm. Medifoam® N has relatively uniform pore size and homogenous morphology with ranged from 100 to 350 μm. Pore size of each dressing was summarized in Table 1.

**Table 1:** Pore size of dressings

	Wound contact layer (μm)	Cross-section (μm)
Medifoam® N	25 ~ 75	100 ~ 350
A	52 ~ 154	169 ~ 455
B	53 ~ 158	241 ~ 366
A1	32 ~ 214	337 ~ 726
L	22 ~ 88	456 ~ 917
P	112 ~ 423	325 ~ 421
S	75 ~ 255	215 ~ 413
C	>1000	177 ~ 346
T	62 ~ 232	216 ~ 378
S1	88 ~ 453	384 ~ 716
M	55 ~ 343	346 ~ 645
P1	65 ~ 348	387 ~ 614

The physical properties of Medifoam® N dressing was compared with other commercially available foam dressings. Description of methods for each of the following analyses, including equations, will be presented below for thickness, density, MVTR, absorption rate, moisture retention capacity, tensile strength, elongation).

**Fig. 2:**
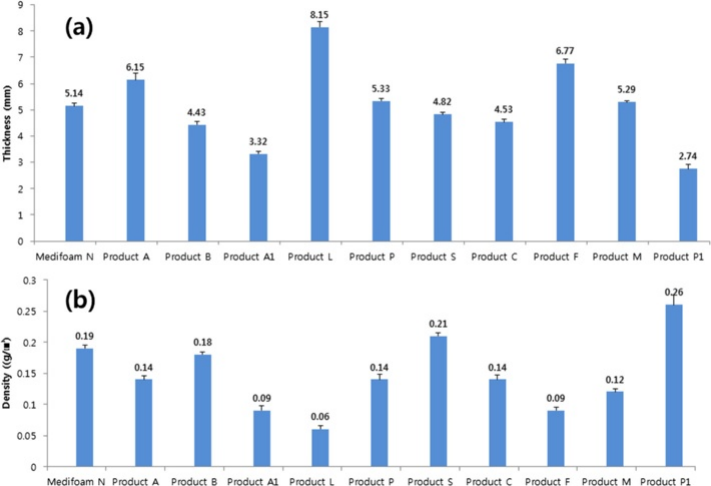
Mean thickness (**a**) and density (**b**) of dressings

Thickness and density are shown in Fig. 2. As shown in Fig. 2a, Medifoam® N has a medium thickness of 5.14 mm and dressing L has highest value in thickness among all dressings. Figure 2(b) shows the density of each dressing. Dressing P1 has the highest density among all tested dressings and Medifoam® N has also an excellent density (0.19 g/cm^3^) compared to other dressings.

**Fig. 3:**
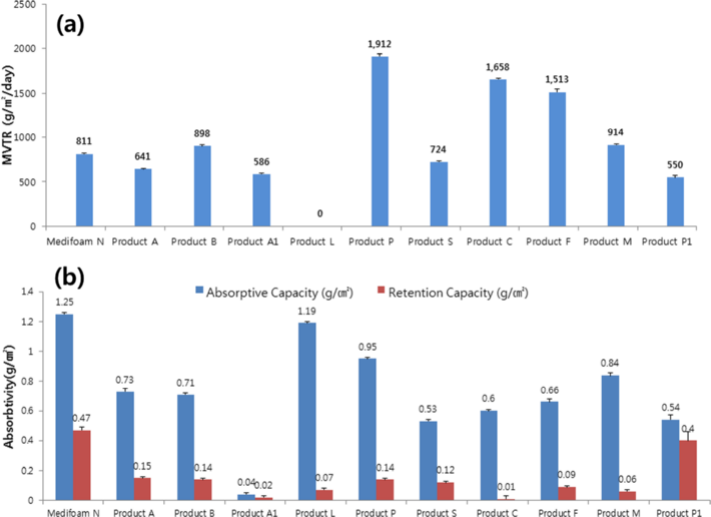
Mean MVTR (**a**) and absorption/retention capacities (**b**) of dressing

Generally, a lower value of MVTR may impede wound healing due to poor drainage of the absorbed exudation. Accordingly, excessively high MVTR values may give rise to dry wound surface due to excessive loss of fluid as water vapor. Thus, MVTR along with absorption/retention capacity is one of the important characteristics for a device to ensure moist wound healing. MVTR and absorption/retention capacity was evaluated as shown in Fig. 3. As shown in Fig. 3a, MVTR of Medifoam® N has a mid-range value among the products (811 g/m^2^/day). Dressing P has the highest value of MVTR and L has the lowest value. The absorption/retention capacity of a dressing is crucial in absorbing exudation and retaining post-absorption moist condition, respectively. As shown in Fig. 3b, absorption/retention capacity of Medifoam® N was notably higher than other dressings, i,e, Medifoam® N has absorption and retention capacity of 1.25 and 0.47 g/cm^2^, respectively.

**Fig. 4:**
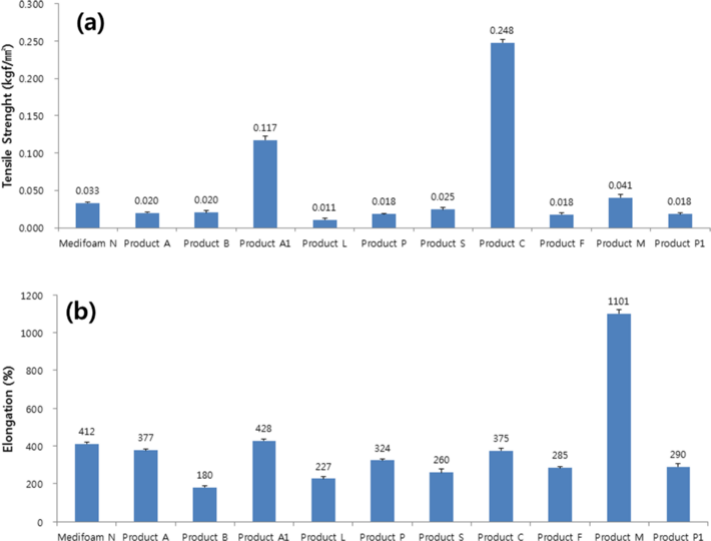
Mean tensile strength (**a**) and elongation (**b**) of dressings

Tensile strength and elongation was shown in Fig. 4. As shown in Fig. 4a, Medifoam® N has good tensile strength (0.033 kgf mm^−2^) with a mid-ranged value. Dressing C has highest tensile strength among all tested dressings. The mean tensility of the other 8 products ranged about 0.01 kgf mm^−2^ ~ 0.025 kgf mm^−2^. As shown in Fig. 4b, dressing M has the greatest elongation properties among all the dressings tested (mean, 1101 %), followed by dressing A1 (428 %). Medifoam® N has also good elongation property (412 %). The remaining 8 products were ranged around 180–377 %.

### In vivo analysis of wound healing

**Fig. 5:**
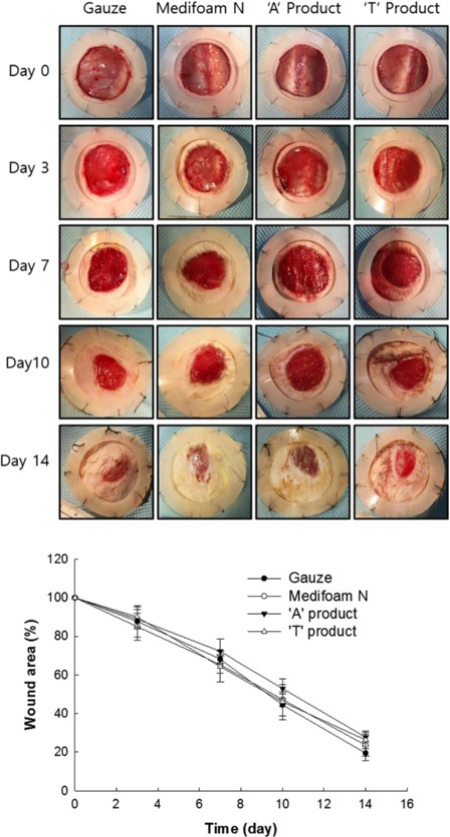
Visual comparison of wound healing using gauze, Medifoam® N, dressing A and T

Wound healing potential of Medifoam® N was studied using rat wound healing model and compared with dressing A and T as shown in Fig. 5. A visual comparison of skin recovery was recorded photographically. Comparisons on wound closure were done day by day. Until day 7, Medifoam® N showed slightly smaller wound area than other treatment even though it was not practically different with other treatment at day 14. At Day 14, no significant difference was observed with respect to evidence of inflammation and rejection, and rate of wound healing (skin recovery). Of the dressing tested, only gauze showed evidence of wound adhesion. On Day 14, specimens from the entire groups were almost cured, with no materials sticking to the wound save for gauze, evidencing effective prevention of secondary skin impairment and pyrogenic, flaring or inflammation as rejection symptoms. For all test groups, the specimens showed fast rate of recovery and approved that the wound dressing materials effectively promoted wound healing.

**Fig. 6:**
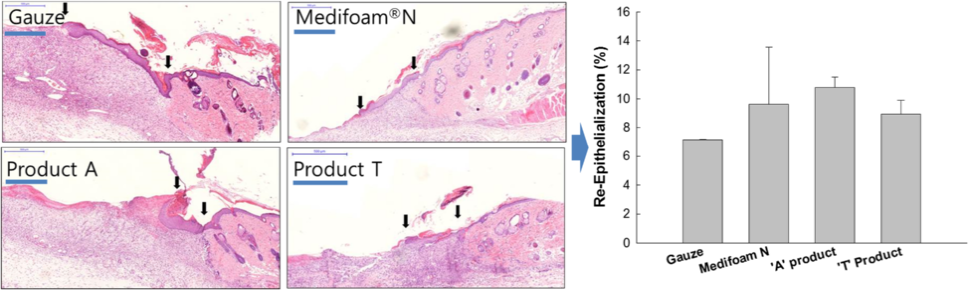
Epidermal Staining (x100, Left photo). Epidermal Recovery Rate (right). Assuming the entire length of wound being ‘C’ and the epidermal recovery rates being ‘a’ and ‘b’ at both ends, the rate of epidermal regeneration were calculated by [(a + b)/C]*100 to represent in percent. (Scale bar = 500 μm)

Figure 6 shows the histopathological analysis of skin tissue samples from wound healing area. As shown in Fig. 6, initial wounded area and new generated epidermal area was marked with arrows at Day 7. Assuming the entire length of wound being ‘C’ and the epidermal recovery rates being ‘a’ and ‘b’ at both ends, the rate of epidermal regeneration were calculated by (a + b)/c for percentage wise represention. No significant difference in re-epithelization was observed between Medifoam® N and competitors’ products.

**Fig. 7:**
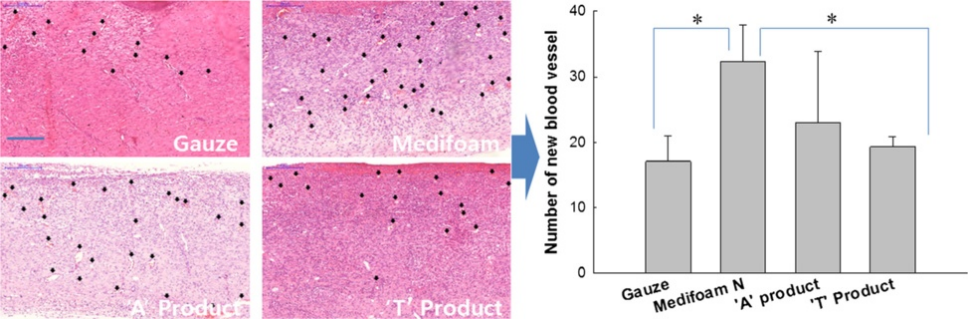
Staining of blood vessels generated (x 200). *: *p* < 0.01. **: *p* < 0.05

**Fig. 8:**
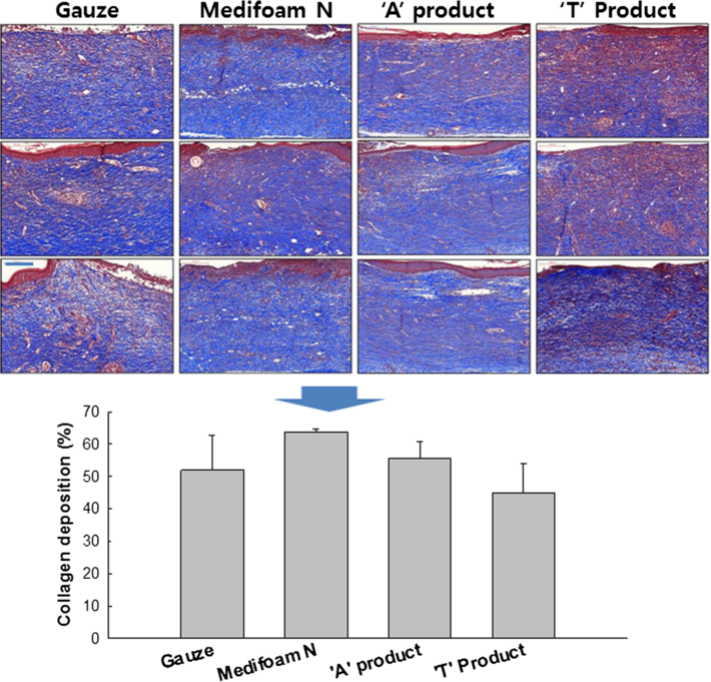
Collagen Deposition on the rat skin tissues. Rat skin was stained with Masson’s Trichrome Staining on Day 14 for collagen staining, followed by measurement of a total of three epidermal areas in confirmation of total recovery of skin tissues by way of collagen deposition rate (x 200). (Scale bar = 200 μm)

Figure 7 shows the blood vessel generation. Blood vessels generated for recovery of impaired skin tissues were marked with arrows, in confirmation of impaired skin tissue regeneration. Compared to other dressings, angiogenic effects of Medifoam®N on day 14 showed relatively higher than treatment with gauze or ‘T’ product. In recovery of wounds, angiogenic effect is one of the most important factors, which is why Medifoam® N is expected to serve an important role in healing wounds with its angiogenic effect. Furthermore, impaired collagen deposition was observed in evaluation of the epidermal connective tissues composed (Fig. 8). Collagen deposition was stained with Masson’s Trichrome Staining on Day 14, followed by measurement of a total of three epidermal areas in confirmation of total recovery of skin tissues by way of collagen deposition rate. All tested groups confirmed collagen deposition with no significant differences, supporting the usefulness of all investigated dressings.

## Discussion

In this study, we compared Medifoam® N with other commercially available polyurethane foam-based dressings and these revealed a unique profile of Medifoam® N. Above all, Medifoam® N, a dressing material for cut surgical wounds, has the smallest pore (25 ~ 75 μm) and cell (100 ~ 350 μm) sizes. The pore size of the wound contact layer is essential to exclude fibroblast and keratin, thus contributing to reduced secondary damage upon dressing change [18]. Additionally, the cell size of absorptive layer may influence absorption capacity for exudate. Leakage of exudate may contaminate clothing or furniture if not retained. These results indicate that the smallest and uniform pore/cell sizes of Medifoam® N can effectively exclude tissue formed and better absorb exudation and maintain moist condition. Furthermore, the smallest pore size of Medifoam®N may contribute strong capillary action. In particular, as discussed above, absorption/retention capacity is regarded as the most important physical properties of dressing materials, i.e. good absorption/retention properties enable to absorb exudates effectively and contained within the dressing to provide a sustained, moderately moist wound environment [13, 19]. Medifoam®N has the densest structure among all the products tested, and demonstrated a remarkable absorption capacity (1.25 g/cm^2^) and excellent retention capacity (0.47 g/cm^2^). Practically, product L and P showed almost similar absorption capacity as well as Medifoam®N, i.e. they have relatively higher porosity with homogeneous pore size. These properties must be influenced to increase absorption capacity. However, their porous structure in cross-section was relatively loose compared to Medifoam®N and these properties must be the reason for the lower retention capacity. For example, surface porosity of product A1 was lower than other product and, pores in cross-section were also large and loose. Therefore, these properties must be induced that product A1 has lower capacity both in absorption and retention. Interestingly, product P1 showed relatively lower apsortion capacity while its retention capacity was similar to Medifoam®N. Since their MVTR value was lower than other product except for product L, it is likely that absorbed exudates were almost retained in the matrix. For example, product C showed second highest in MVTR value and this value must be affected to absorption/retention capacity, i.e. absorption capacity of product C was practically similar to product P1 but its retention capacity was significantly smaller than product P1 or Medifoam®N. Furthermore, absorption capacity of product L was practically similar to Medifoam®N but its retention capacity was less than half. Thomas studied absorption and retention capacities of various foam-film dressings, and he reported that foam-film dressings have a wide variation in product performance with differences in retention capacities. This was likely to be attributable in part to varying moisture permeabilities of the outer film layer [20]. In our study of absorption patterns, Medifoam® N rapidly absorbed fluid without substantial horizontal spread (Additional file 1) while other product such as product A did not immediately absorb the solution and some of them was leaked out. These results indicated that Medifoam® N has excellent physical properties as a wound healing material. Rapid vertical exudate absorption and retention may help to minimize the risk of maceration of skin around the wound periphery. This benefit is likely to be enhanced by Medifoam® N’s moderate MVTR, which suggests an ability to allow for an optimized exudate drainage through evaporation. These properties suggest that Medifoam® N is highly suitable for use in wound management, particularly in wounds with moderate to high production of exudate.

The properties of Medifoam® N which encourage a moist wound healing environment may help to reduce the frequency of dressing changes, mitigating not only healthcare costs but also patient pain and inconvenience. However, one concern with infrequent dressing changes is the potential for ingrowth of new tissue into the dressing over time, which may result in shearing trauma with inevitable body movement upon dressing removal.

Our animal study illustrated the tendency toward dressing-wound adhesion with traditional gauze dressings. Our study demonstrated Medifoam® N’s remarkably small pore size (51.3 μm) and high pore size uniformity. This has substantial clinical significance with regards to wound-dressing adhesion, particularly when less frequent dressing changes are necessary. A dressing with a small enough pore size is able to exclude newly formed tissue and keratin during the healing process, precluding tissue ingrowth. An in vitro study conducted in three types of cultured fibroblasts showed that, after 3 h of culture on a substrate plane, the average new fibroblast length was approximately 50 μm [18]. They reported that fibroblasts grew in size in a nearly linear fashion to reach a peak length of approximately 140 μm after 24 h. The small pore size of Medifoam® N, and possibly also Dressing L, could thus be expected to prevent the entry of fibroblasts into the dressing material. Indeed, our animal study showed no dressing adhesion with Medifoam® N or the other two polyurethane foam dressings during wound healing. Consistent with this finding, previous clinical studies of the postoperative use of Medifoam® N have also demonstrated no detachment of the re-epithelialized tissue layer upon dressing removal after 24–48 h and again after Days 3, 6, and 9 [21]. A recent animal study also suggested that pore size of polyurethane foam dressing plays a crucial role in wound healing [22]. Using polyurethane foam dressings under negative pressure, the investigators confirmed that pore size was positively associated with tissue ingrowth into the dressing interface; compared with foams of small diameter pore size, the extent of tissue ingrowth was 3.7-fold higher with foams of medium pore size, and 5.6-fold higher with foams of large pore size on day 7 of healing. In addition, larger pore sizes resulted in greater wound deformation and thickness of granulation tissue formation [22]. The potential for ingrowth of newly formed tissue at the dressing-wound interface is thus a key consideration in wound dressing selection – particularly when dressings which promote a moist wound environment are used with the intention of extending the period of time between dressing changes.

Medifoam® N has mid-range levels of thickness and tensile strength in our physical properties study, with excellent elongation properties as shown in Figs. 2 and 4, suggesting a good balance of sturdiness and flexibility, which is an important feature of dressing materials to enhance biomechanical protection of the wound area [5, 14, 17]. In our animal study, no practical visual differences in wound healing rates and angiogenesis rate were observed between Medifoam® N and either gauze, dressing A, or dressing T (Fig. 6). The rate of angiogenesis is strongly influenced by an initially hypoxic environment, such as that provided by an occlusive dressing, and that angiogenesis plays a key role in wound healing [23]. The optimal wound healing environment facilitated by the advanced core physical properties of Medifoam® N, discussed above, has been illustrated in other in vivo studies. For example, in another murine model, Medifoam® N showed superior wound-closure and re-epithelialization properties versus other polyurethane foam dressings when applied to full-thickness wounds of 5 mm diameter [24]. The enhancement of the wound healing process with Medifoam® N versus traditional Vaseline gauze has also been demonstrated in clinical studies [25]. Among 120 hospitalized burn patients who had undergone skin graft surgery, those wounds to which Medifoam® N were applied showed faster healing times (*P* < 0.01), less wound pain (*P* < 0.01) and less scar formation compared with those to which Vaseline gauze was applied [25]. Taken together, the favorable performance of Medifoam® N across our in vitro and in vivo studies with regard to the creation of an optimal wound healing environment suggests it is a favorable choice for wound treatment in the clinical setting, according to its commercial indications. Our studies support other preclinical and clinical studies that suggest excellent facilitation of wound healing with this product and may provide more insight to the clinical observations made then [24, 25]. In fact, the clinical data collected in these studies showed a bigger difference to other foams than the rat study conducted by us.

## Conclusion

We compared eleven commercially available polyurethane foam dressings. The physicochemical testing provided insight into key differences of devices of the same category. Among them, Medifoam® N has the smallest pore and cell sizes with excellent uniformity. It also has optimized physical properties such as thickness, density, tensile strength, elongation, MVTR and adsorptivity. Due to these characteristics, Medifoam® N showed excellent absorption/retention capacity and fluid absorption patterns. Furthermore, these also contributed wound healing potential observed in our study and previously [24, 25]. We found that Medifoam® N showed the best results among the employed dressing samples. We found that Medifoam® N showed the best results among the employed dressing samples.

## Abbreviations

ASTM, American Society for Testing and Material; EN, European Norm; FE-SEM, field-emission scanning electron microscope; H&E, hematoxylin and eosin; MVTR, moisture-vapor transmission rate; PBS, phosphate-buffered saline; PU, polyurethane; SD, Sprague Dawley

## Additional file

Additional file 1: The absorption pattern observed with Medifoam®N and dressing A. (DOCX 1518 kb)
