# Erratum to: Physical, morphological, and wound healing properties of a polyurethane foam-film dressing

**DOI:** 10.1186/s40824-016-0084-0

**Published:** 2016-12-05

**Authors:** Seung Moon Lee, Il Kyu Park, Yong Soo Kim, Hyun Jung Kim, Hanlim Moon, Stefan Mueller, Young-IL Jeong

**Affiliations:** 1 Genewel Co. Ltd, Gyeonggi-do, Korea; 2 Mundipharma Pte. Ltd, Singapore, Singapore; 3 Biomedical Research Institute, Pusan National University Hospital, 179 Gudeok-ro, Seo-gu, Busan, 602-739 Republic of Korea

## Erratum

The original article contains two errors [1] in the **Materials** sub-section of the **Methods** section, both of which are clarified here:

1) “A, B, A1, L, P, S, C, F, T, M, P1” should read “A, B, A1, L, P, S, C, F, T, S1, M, P1”.

2) “Tegaderm (3 M Co.)” should read “Tielle (J&J), Schaumaverband (3 M Co.)”.
