# Supplementary material for: Physical, morphological, and wound healing properties of a polyurethane foam-film dressing
Source: Biomater Res. 2016 Jun 4;20:15. doi: 10.1186/s40824-016-0063-5 (PMC4893288; doi:10.1186/s40824-016-0063-5)
Supplement: Additional file 1: — The absorption pattern observed with Medifoam®N and dressing A. (DOCX 1518 kb) [file 40824_2016_63_MOESM1_ESM.docx]

**Supplementary results**

**Methods**

***In vitro* fluid absorption patterns**

To measure absorption patterns, PBS solution containing pigment was used. After that, 10 g of PBS solution containing pigment was dropped onto the dressings at the height of 1 cm and then left for 5 min. After that, fluid absorption into the foam was assessed.

**Results**

**In vitro absorption patterns**

Figure 5 shows the in vitro absorption patterns of Medifoam^®^ N compared with dressing A. As shown in Figure 5, all dressings rapidly absorbed PBS solution containing pigment. However, absorption pattern between dressing A and Medifoam^®^ N was quite different, i.e. PBS solution was rapidly absorbed into Medifoam^®^ N without horizontal spread. In contrast, dressing A did not immediately absorb the solution and the absorption pattern was far less uniform. Furthermore, some of PBS solution was not absorbed into dressings and then leaked out. These results indicated that Medifoam^®^ N has excellent physical properties as a wound healing material.


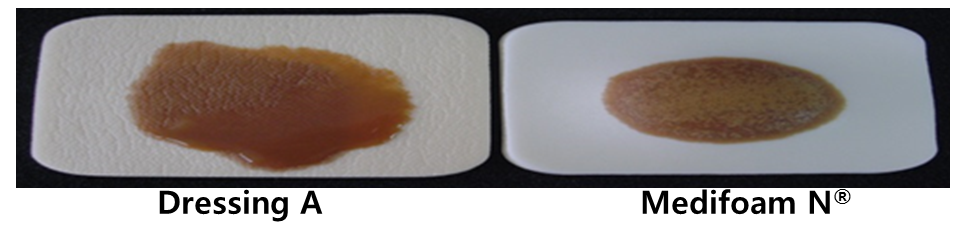


Figure s1. The absorption pattern observed with Medifoam^®^N and dressing A.


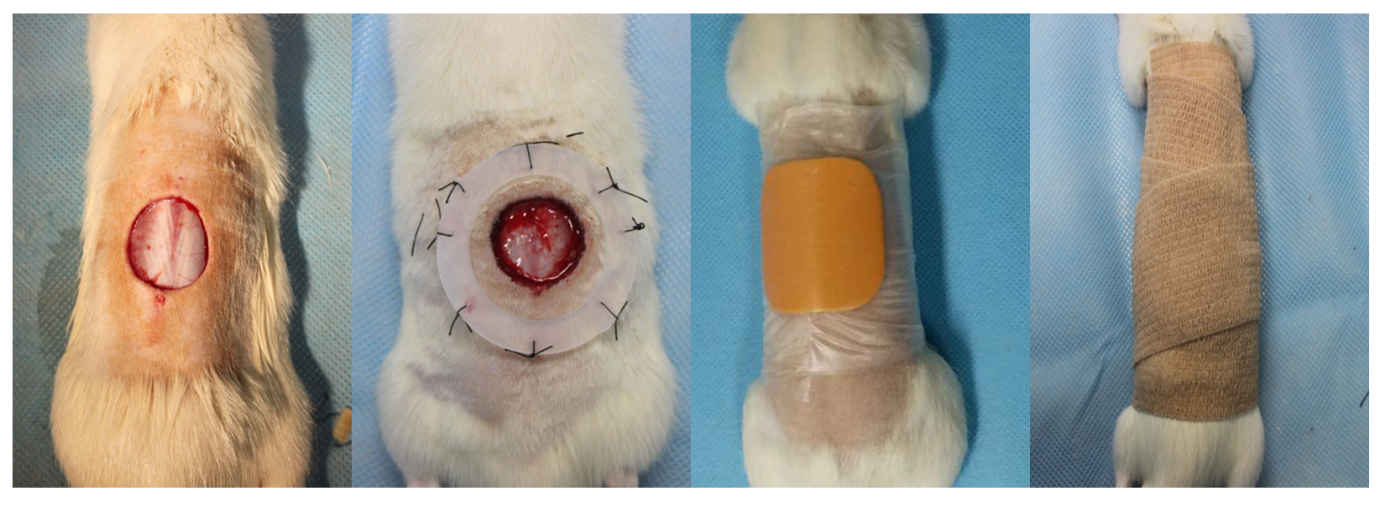


Figure s2. Procedures of wound healing experiment. The backs of rats were shaved and then circular wounds with 25mm diameter induced using a blade. Wounds were treated by foam dressings and then covered with gauze to avoid contamination, and secured with a film dressing (polyurethane film, Opsite, Smith & Nephew, UK). Dressings were bandaged to prevent nibbling by rats and replaced on 2 to 3-day intervals.
